# 3-(Ferrocen-1-ylcarbon­yl)-1-methyl-4-(4-methyl­phen­yl)spiro­[pyrrolidine-2,11′-indeno­[1,2-*b*]quinoxaline]

**DOI:** 10.1107/S1600536812036951

**Published:** 2012-09-15

**Authors:** B. Vijayakumar, D. Gavaskar, T. Srinivasan, R. Raghunathan, D. Velmurugan

**Affiliations:** a Centre of Advanced Study in Crystallography and Biophysics, University of Madras, Maraimalai (Guindy) Campus, Chennai 600 025, India; b Department of Organic Chemistry, University of Madras, Maraimalai (Guindy) Campus, Chennai 600 025, India

## Abstract

In the title compound, [Fe(C_5_H_5_)(C_32_H_26_N_3_O)], the pyrrolidine ring adopts a twist conformation. The indeno–quinoxaline ring system [86.44 (5)°], the methyl­phenyl ring [86.06 (7)°] and the ferrocene rings [82.00 (7) and 83.95 (9)°] are almost perpendicular to the pyrrolidine ring. The two cyclopentadienyl rings adopt an eclipsed conformation. The crystal structure features C—H⋯N inter­actions.

## Related literature

For the biological activity of ferrocene derivatives, see: Jaouen *et al.* (2004 ▶); Biot *et al.* (2004 ▶); Fouda *et al.* (2007 ▶). For related structures, see: Satis Kumar *et al.*(2007 ▶); Kamala *et al.* (2009 ▶); Gunasekaran *et al.* (2010 ▶); For puckering and asymmetry parameters, see: Cremer & Pople (1975 ▶); Nardelli (1983 ▶).
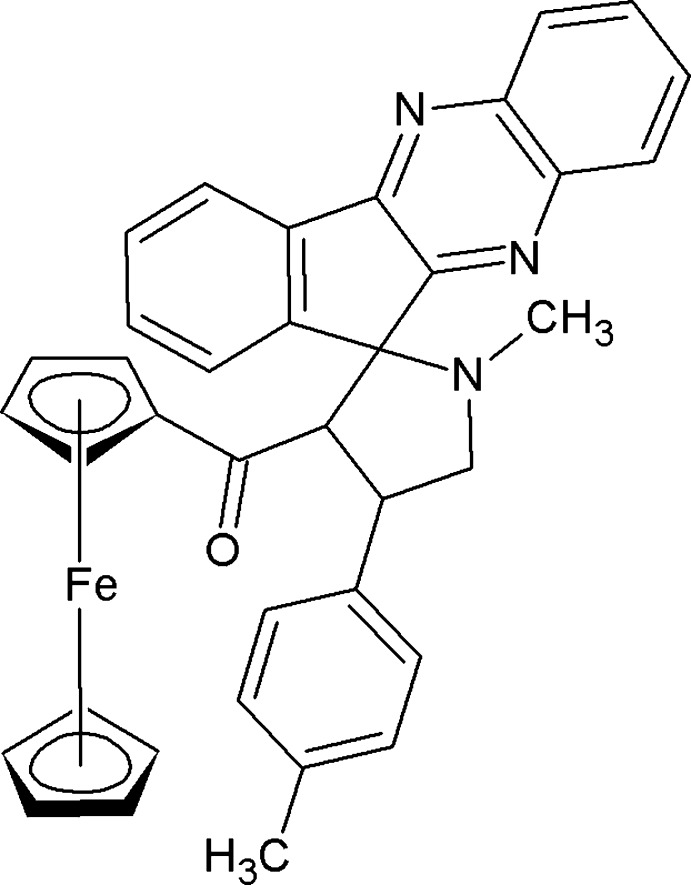


## Experimental

### 

#### Crystal data

[Fe(C_5_H_5_)(C_32_H_26_N_3_O)]
*M* *_r_* = 589.50
Monoclinic, 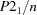

*a* = 11.5966 (3) Å
*b* = 11.8658 (3) Å
*c* = 20.9383 (6) Å
β = 90.616 (2)°
*V* = 2881.01 (13) Å^3^
*Z* = 4
Mo *K*α radiation
μ = 0.56 mm^−1^
*T* = 293 K
0.2 × 0.2 × 0.2 mm

#### Data collection

Bruker SMART APEXII area-detector diffractometer
26509 measured reflections
7145 independent reflections
5247 reflections with *I* > 2σ(*I*)
*R*_int_ = 0.026

#### Refinement

*R*[*F* ^2^ > 2σ(*F* ^2^)] = 0.037
*wR*(*F* ^2^) = 0.110
*S* = 1.02
7145 reflections
381 parameters
H-atom parameters constrained
Δρ_max_ = 0.26 e Å^−3^
Δρ_min_ = −0.31 e Å^−3^

### 

Data collection: *APEX2* (Bruker, 2008 ▶); cell refinement: *SAINT* (Bruker, 2008 ▶); data reduction: *SAINT*; program(s) used to solve structure: *SHELXS97* (Sheldrick, 2008 ▶); program(s) used to refine structure: *SHELXL97* (Sheldrick, 2008 ▶); molecular graphics: *ORTEP-3* (Farrugia, 1997 ▶); software used to prepare material for publication: *SHELXL97* and *PLATON* (Spek, 2009 ▶).

## Supplementary Material

Crystal structure: contains datablock(s) global, I. DOI: 10.1107/S1600536812036951/bt6825sup1.cif

Structure factors: contains datablock(s) I. DOI: 10.1107/S1600536812036951/bt6825Isup2.hkl

Supplementary material file. DOI: 10.1107/S1600536812036951/bt6825Isup4.mol

Additional supplementary materials:  crystallographic information; 3D view; checkCIF report

## Figures and Tables

**Table 1:** Hydrogen-bond geometry (Å, °)

*D*—H⋯*A*	*D*—H	H⋯*A*	*D*⋯*A*	*D*—H⋯*A*
C28—H28⋯N3^i^	0.93	2.49	3.416 (2)	176

Symmetry code: (i) 
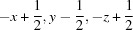
.

**Fig. 1.**
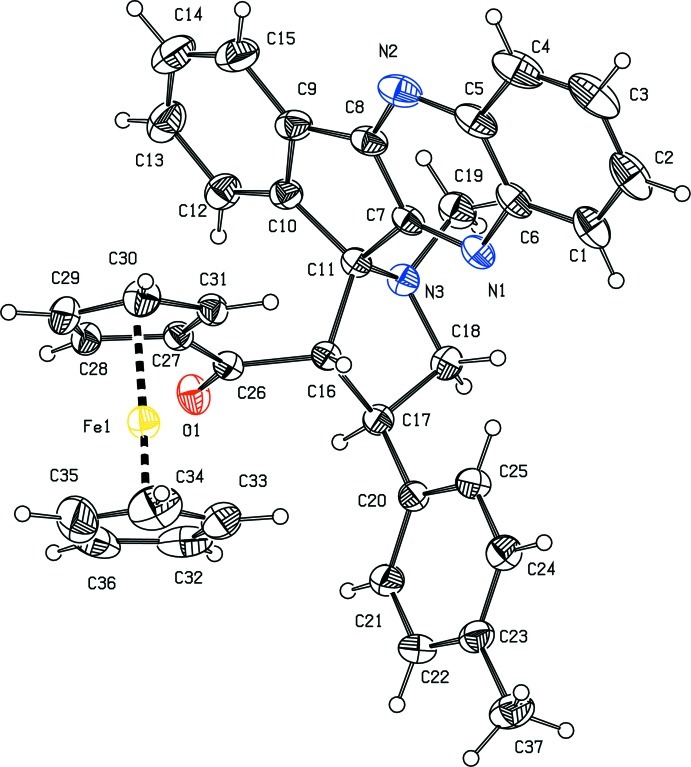
Molecular structure of the title compound, showing displacement ellipsoids drawn at the 30% probability level. H atoms are presented as a small spheres of arbitrary radius.

**Fig. 2.**
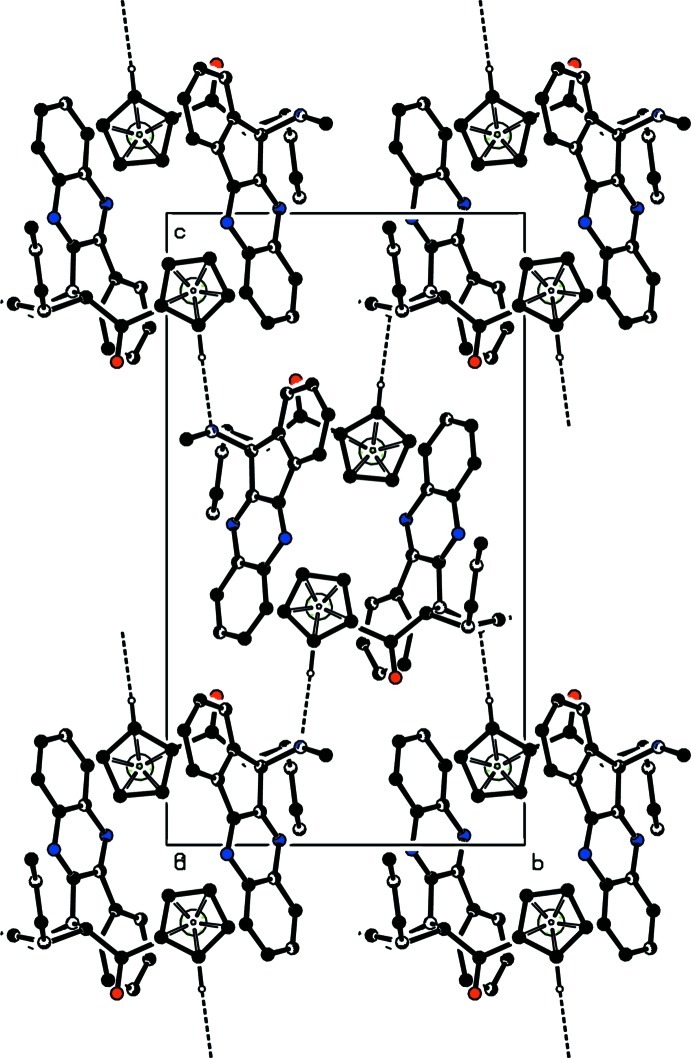
The molecular packing viewed down the a axis. Dashed lines shows the intermolecular C–H···N hydrogen bonds.

## supplementary crystallographic information

### Comment

Ferrocene attached compounds have prominent biological activities like antitumor (Jaouen *et al.*, 2004), antimalarial and antifungal (Biot *et al.*, 2004), and antibacterial (Fouda *et al.*, 2007) and find applications in medicinal chemistry. In this background, X-ray study of the title compound was carried out to obtain the information of molecular conformations and crystal packing in the solid state.

X-ray analysis confirms the molecular structure and atom connectivity as illustrated in Fig. 1. The bond lengths and angles of titled compound agree with those observed in other ferrocene derivative 4'-Ferrocenyl-3'- 4-methoxybenzoyl)-1'-methylspiro[1*H*-indole-3(2*H*), 2'-pyrrolidin]-2-one (Satis Kumar *et al.*, 2007). The dihedral angle between indeno-quinoxaline ring system and the pyrrolidine ring is 87.84 (7)° and the dihedral angle between pyrrolidine ring and the phenyl ring is 86.06 (9) °. The dihedral angle between pyrrolidine ring and the ferrocene rings are 81.00 (10)° and 83.94 (12)°. This clearly shows that the indeno-quinoxaline ring system, the phenyl and ferrocene rings are almost perpendicular to the pyrrolidine ring.

The pyrrolidine ring adopts a twisted conformation with the puckering parameters q_2_ and φ (Cremer & Pople, 1975) and the smallest displacement asymmetric parameters, δ, (Nardelli, 1983) as follows: q_2_ = 0.3858 (16) Å, φ = 343.1 (3)° and Δ_2_ (C16) = 2.13 (15)°. In the ferrocene, Cg3 and Cg4 are the centroids of the C27—C31 and C32—C36 rings, respectively. Fe1—Cg3 and Fe1—Cg4 distances are 1.6430 (8) and 1.6495 (10) Å, respectively and the Cg3—Fe1—Cg4 angle is 177.74 (6)°. In addition to the van der Waals interactions, the crystal packing is stabilized by C—H···N interaction (Table. 1 & Fig. 2).

### Experimental

Ninhydrin (1 mM) and 1, 2-phenylenediamine (1 mM) were mixed and stirred with 10mL of methanol for 10 min. To this mixture 1 mM of Sarcosine and 1 mM of ferrocene derived dipolarophile were added and was refluxed up to the end of the reaction as observed by TLC. The solvent content from the mixture was removed under reduced pressure and the crude product was obtained. Using column chromatography the crude extract was purified by 4:1 ratio of petroleum ether and ethyl acetate. Finally, single crystals suitable for the X-ray diffraction were obtained by slow evaporation at room temperature.

### Refinement

Hydrogen atoms were placed in calculated positions with C—H ranging from = 0.93Å to 0.98Å and refined using the riding model approximation with *U*_iso_(H) = 1.2 *U*_eq_(C) or *U*_iso_(H) = 1.5 *U*_eq_(C_methyl_).

### Crystal data

**Table:**

[Fe(C_5_H_5_)(C_32_H_26_N_3_O)]	*F*(000) = 1232
*M**_r_* = 589.50	*D*_x_ = 1.359 Mg m^−^^3^
Monoclinic, *P*2_1_/*n*	Mo *K*α radiation, λ = 0.71073 Å
Hall symbol: -P 2yn	Cell parameters from 7145 reflections
*a* = 11.5966 (3) Å	θ = 2.0–28.3°
*b* = 11.8658 (3) Å	µ = 0.56 mm^−^^1^
*c* = 20.9383 (6) Å	*T* = 293 K
β = 90.616 (2)°	Block, brown
*V* = 2881.01 (13) Å^3^	0.2 × 0.2 × 0.2 mm
*Z* = 4	

### Data collection

**Table:**

Bruker SMART APEXII area-detector diffractometer	5247 reflections with *I* > 2σ(*I*)
Radiation source: fine-focus sealed tube	*R*_int_ = 0.026
Graphite monochromator	θ_max_ = 28.3°, θ_min_ = 2.0°
ω and φ scans	*h* = −14→15
26509 measured reflections	*k* = −15→14
7145 independent reflections	*l* = −27→25

### Refinement

**Table:**

Refinement on *F*^2^	Primary atom site location: structure-invariant direct methods
Least-squares matrix: full	Secondary atom site location: difference Fourier map
*R*[*F*^2^ > 2σ(*F*^2^)] = 0.037	Hydrogen site location: inferred from neighbouring sites
*wR*(*F*^2^) = 0.110	H-atom parameters constrained
*S* = 1.02	*w* = 1/[σ^2^(*F*_o_^2^) + (0.0573*P*)^2^ + 0.5923*P*] where *P* = (*F*_o_^2^ + 2*F*_c_^2^)/3
7145 reflections	(Δ/σ)_max_ = 0.023
381 parameters	Δρ_max_ = 0.26 e Å^−^^3^
0 restraints	Δρ_min_ = −0.31 e Å^−^^3^

### Special details

**Table:**

Geometry. All esds (except the esd in the dihedral angle between two l.s. planes) are estimated using the full covariance matrix. The cell esds are taken into account individually in the estimation of esds in distances, angles and torsion angles; correlations between esds in cell parameters are only used when they are defined by crystal symmetry. An approximate (isotropic) treatment of cell esds is used for estimating esds involving l.s. planes.
Refinement. Refinement of F^2^ against ALL reflections. The weighted R-factor wR and goodness of fit S are based on F^2^, conventional R-factors R are based on F, with F set to zero for negative F^2^. The threshold expression of F^2^ > 2sigma(F^2^) is used only for calculating R-factors(gt) etc. and is not relevant to the choice of reflections for refinement. R-factors based on F^2^ are statistically about twice as large as those based on F, and R- factors based on ALL data will be even larger.

### Fractional atomic coordinates and isotropic or equivalent isotropic displacement parameters (Å^2^)

**Table:**

	*x*	*y*	*z*	*U*_iso_*/*U*_eq_	
C1	0.29739 (19)	0.35976 (18)	−0.10411 (9)	0.0570 (5)	
H1	0.2331	0.4050	−0.0976	0.068*	
C2	0.3437 (2)	0.3508 (2)	−0.16349 (10)	0.0716 (7)	
H2	0.3110	0.3904	−0.1975	0.086*	
C3	0.4399 (2)	0.2825 (2)	−0.17355 (11)	0.0776 (8)	
H3	0.4719	0.2789	−0.2141	0.093*	
C4	0.4876 (2)	0.2215 (2)	−0.12547 (11)	0.0718 (7)	
H4	0.5502	0.1747	−0.1333	0.086*	
C5	0.44203 (16)	0.22899 (17)	−0.06319 (9)	0.0527 (5)	
C6	0.34719 (15)	0.30038 (15)	−0.05269 (8)	0.0461 (4)	
C7	0.35581 (13)	0.26030 (14)	0.05261 (8)	0.0378 (3)	
C8	0.45054 (14)	0.18715 (15)	0.04184 (9)	0.0447 (4)	
C9	0.49029 (15)	0.14277 (15)	0.10323 (9)	0.0473 (4)	
C10	0.42069 (14)	0.18700 (14)	0.15109 (8)	0.0412 (4)	
C11	0.32625 (13)	0.26329 (13)	0.12383 (7)	0.0357 (3)	
C12	0.44572 (16)	0.16378 (16)	0.21433 (9)	0.0524 (4)	
H12	0.4015	0.1947	0.2467	0.063*	
C13	0.53779 (19)	0.09358 (19)	0.22876 (12)	0.0669 (6)	
H13	0.5544	0.0764	0.2712	0.080*	
C14	0.6054 (2)	0.0487 (2)	0.18112 (13)	0.0735 (7)	
H14	0.6666	0.0016	0.1919	0.088*	
C15	0.58319 (18)	0.07298 (18)	0.11806 (12)	0.0659 (6)	
H15	0.6292	0.0435	0.0860	0.079*	
C16	0.19757 (12)	0.22576 (13)	0.13323 (7)	0.0335 (3)	
H16	0.1661	0.2027	0.0916	0.040*	
C17	0.13242 (14)	0.33248 (14)	0.15504 (8)	0.0384 (4)	
H17	0.1192	0.3268	0.2011	0.046*	
C18	0.21749 (15)	0.42714 (14)	0.14355 (8)	0.0414 (4)	
H18A	0.2111	0.4552	0.1001	0.050*	
H18B	0.2053	0.4890	0.1730	0.050*	
C19	0.42792 (17)	0.44471 (16)	0.14190 (10)	0.0530 (5)	
H19A	0.4262	0.4674	0.0979	0.080*	
H19B	0.4974	0.4032	0.1505	0.080*	
H19C	0.4257	0.5103	0.1687	0.080*	
C20	0.01671 (14)	0.34623 (14)	0.12153 (8)	0.0395 (4)	
C21	−0.08575 (16)	0.34224 (17)	0.15446 (9)	0.0530 (5)	
H21	−0.0841	0.3338	0.1986	0.064*	
C22	−0.19103 (16)	0.35058 (18)	0.12277 (11)	0.0594 (5)	
H22	−0.2587	0.3457	0.1461	0.071*	
C23	−0.19783 (15)	0.36583 (15)	0.05778 (10)	0.0501 (4)	
C24	−0.09542 (16)	0.37090 (16)	0.02483 (9)	0.0500 (4)	
H24	−0.0974	0.3811	−0.0192	0.060*	
C25	0.01009 (15)	0.36115 (15)	0.05584 (8)	0.0457 (4)	
H25	0.0776	0.3647	0.0323	0.055*	
C26	0.18900 (13)	0.12623 (14)	0.17858 (7)	0.0352 (3)	
C27	0.20428 (13)	0.01427 (14)	0.15034 (7)	0.0357 (3)	
C28	0.20015 (15)	−0.08985 (14)	0.18424 (9)	0.0429 (4)	
H28	0.1963	−0.0982	0.2283	0.051*	
C29	0.20304 (17)	−0.17801 (16)	0.13901 (10)	0.0535 (5)	
H29	0.2013	−0.2547	0.1482	0.064*	
C30	0.20898 (17)	−0.13008 (16)	0.07734 (9)	0.0527 (5)	
H30	0.2115	−0.1699	0.0391	0.063*	
C31	0.21043 (14)	−0.01244 (14)	0.08348 (8)	0.0408 (4)	
H31	0.2146	0.0392	0.0502	0.049*	
C32	−0.07832 (18)	0.0154 (3)	0.14632 (14)	0.0808 (8)	
H32	−0.0782	0.0858	0.1659	0.097*	
C33	−0.07821 (18)	−0.0055 (2)	0.08054 (12)	0.0733 (7)	
H33	−0.0779	0.0487	0.0485	0.088*	
C34	−0.0786 (2)	−0.1200 (3)	0.07176 (14)	0.0866 (8)	
H34	−0.0791	−0.1567	0.0325	0.104*	
C35	−0.0781 (2)	−0.1716 (3)	0.1306 (2)	0.0998 (11)	
H35	−0.0775	−0.2490	0.1378	0.120*	
C36	−0.0786 (2)	−0.0890 (4)	0.17706 (14)	0.0946 (10)	
H36	−0.0790	−0.1008	0.2210	0.114*	
C37	−0.31185 (17)	0.3780 (2)	0.02292 (12)	0.0678 (6)	
H37A	−0.3713	0.3418	0.0470	0.102*	
H37B	−0.3068	0.3433	−0.0184	0.102*	
H37C	−0.3299	0.4565	0.0179	0.102*	
N1	0.30156 (12)	0.31494 (12)	0.00747 (6)	0.0426 (3)	
N2	0.49441 (14)	0.16877 (15)	−0.01466 (8)	0.0561 (4)	
N3	0.32874 (11)	0.37402 (11)	0.15487 (6)	0.0384 (3)	
O1	0.17339 (11)	0.13915 (11)	0.23535 (5)	0.0502 (3)	
Fe1	0.06387 (2)	−0.07469 (2)	0.123016 (11)	0.04181 (9)	

### Atomic displacement parameters (Å^2^)

**Table:**

	*U*^11^	*U*^22^	*U*^33^	*U*^12^	*U*^13^	*U*^23^
C1	0.0695 (13)	0.0608 (12)	0.0408 (10)	−0.0229 (10)	0.0043 (9)	−0.0020 (9)
C2	0.0903 (17)	0.0848 (17)	0.0399 (11)	−0.0422 (14)	0.0044 (10)	−0.0026 (11)
C3	0.0872 (17)	0.103 (2)	0.0433 (12)	−0.0423 (15)	0.0236 (11)	−0.0244 (13)
C4	0.0661 (14)	0.0919 (18)	0.0579 (14)	−0.0252 (13)	0.0249 (11)	−0.0307 (13)
C5	0.0510 (11)	0.0600 (12)	0.0476 (10)	−0.0217 (9)	0.0151 (8)	−0.0189 (9)
C6	0.0517 (10)	0.0489 (10)	0.0380 (9)	−0.0218 (8)	0.0080 (7)	−0.0086 (7)
C7	0.0373 (8)	0.0370 (8)	0.0393 (9)	−0.0097 (7)	0.0073 (6)	−0.0064 (7)
C8	0.0378 (9)	0.0427 (9)	0.0537 (10)	−0.0075 (7)	0.0089 (7)	−0.0114 (8)
C9	0.0369 (9)	0.0424 (10)	0.0628 (12)	−0.0022 (7)	0.0026 (8)	−0.0088 (8)
C10	0.0355 (8)	0.0385 (9)	0.0496 (10)	−0.0034 (7)	−0.0016 (7)	−0.0036 (7)
C11	0.0356 (8)	0.0368 (8)	0.0348 (8)	−0.0032 (6)	0.0010 (6)	−0.0023 (6)
C12	0.0497 (10)	0.0536 (11)	0.0538 (11)	0.0018 (8)	−0.0090 (8)	−0.0014 (9)
C13	0.0622 (13)	0.0615 (13)	0.0765 (15)	0.0061 (10)	−0.0222 (11)	0.0043 (11)
C14	0.0544 (13)	0.0641 (14)	0.1016 (19)	0.0161 (11)	−0.0182 (12)	−0.0036 (13)
C15	0.0484 (11)	0.0575 (13)	0.0917 (18)	0.0102 (9)	0.0034 (11)	−0.0122 (11)
C16	0.0339 (8)	0.0361 (8)	0.0305 (8)	−0.0021 (6)	0.0011 (6)	−0.0018 (6)
C17	0.0409 (9)	0.0408 (9)	0.0335 (8)	0.0033 (7)	0.0032 (6)	−0.0011 (7)
C18	0.0475 (9)	0.0357 (9)	0.0410 (9)	0.0025 (7)	−0.0014 (7)	−0.0051 (7)
C19	0.0515 (11)	0.0469 (10)	0.0606 (12)	−0.0133 (8)	−0.0008 (9)	−0.0063 (9)
C20	0.0394 (8)	0.0360 (8)	0.0432 (9)	0.0020 (7)	0.0048 (7)	0.0002 (7)
C21	0.0468 (10)	0.0617 (12)	0.0506 (11)	0.0095 (9)	0.0104 (8)	0.0096 (9)
C22	0.0385 (10)	0.0653 (13)	0.0747 (14)	0.0055 (9)	0.0139 (9)	0.0093 (11)
C23	0.0394 (9)	0.0400 (10)	0.0709 (13)	0.0003 (7)	−0.0035 (8)	−0.0021 (9)
C24	0.0492 (10)	0.0511 (11)	0.0497 (10)	−0.0014 (8)	−0.0039 (8)	−0.0026 (8)
C25	0.0383 (9)	0.0532 (11)	0.0456 (10)	−0.0015 (8)	0.0033 (7)	−0.0006 (8)
C26	0.0337 (8)	0.0400 (9)	0.0318 (8)	−0.0031 (6)	0.0002 (6)	−0.0008 (6)
C27	0.0333 (8)	0.0392 (9)	0.0347 (8)	−0.0015 (6)	−0.0013 (6)	0.0011 (6)
C28	0.0423 (9)	0.0427 (9)	0.0436 (9)	0.0008 (7)	−0.0034 (7)	0.0065 (7)
C29	0.0602 (12)	0.0362 (10)	0.0641 (12)	0.0060 (8)	0.0006 (9)	0.0032 (8)
C30	0.0615 (12)	0.0460 (11)	0.0508 (11)	0.0047 (9)	0.0035 (9)	−0.0112 (8)
C31	0.0436 (9)	0.0427 (9)	0.0362 (8)	0.0006 (7)	0.0044 (7)	0.0006 (7)
C32	0.0390 (11)	0.099 (2)	0.105 (2)	0.0058 (12)	0.0031 (12)	−0.0299 (17)
C33	0.0480 (12)	0.0911 (18)	0.0804 (16)	0.0039 (11)	−0.0125 (11)	0.0230 (14)
C34	0.0658 (15)	0.100 (2)	0.093 (2)	−0.0099 (14)	−0.0330 (14)	−0.0246 (17)
C35	0.0642 (16)	0.0845 (19)	0.150 (3)	−0.0357 (14)	−0.0271 (18)	0.036 (2)
C36	0.0426 (12)	0.167 (3)	0.0742 (17)	−0.0171 (16)	0.0051 (11)	0.032 (2)
C37	0.0438 (11)	0.0635 (13)	0.0959 (17)	0.0014 (10)	−0.0121 (10)	−0.0107 (12)
N1	0.0465 (8)	0.0462 (8)	0.0352 (7)	−0.0094 (6)	0.0064 (6)	−0.0033 (6)
N2	0.0477 (9)	0.0622 (10)	0.0588 (10)	−0.0089 (8)	0.0153 (7)	−0.0201 (8)
N3	0.0403 (7)	0.0357 (7)	0.0392 (7)	−0.0039 (6)	−0.0005 (6)	−0.0054 (6)
O1	0.0692 (8)	0.0498 (7)	0.0319 (6)	−0.0028 (6)	0.0060 (5)	−0.0001 (5)
Fe1	0.04165 (15)	0.04181 (15)	0.04185 (15)	−0.00598 (10)	−0.00464 (10)	0.00020 (10)

### Geometric parameters (Å, º)

**Table:**

C1—C2	1.364 (3)	C20—C25	1.388 (2)
C1—C6	1.406 (3)	C21—C22	1.387 (3)
C1—H1	0.9300	C21—H21	0.9300
C2—C3	1.398 (4)	C22—C23	1.374 (3)
C2—H2	0.9300	C22—H22	0.9300
C3—C4	1.353 (4)	C23—C24	1.381 (3)
C3—H3	0.9300	C23—C37	1.510 (3)
C4—C5	1.415 (3)	C24—C25	1.384 (2)
C4—H4	0.9300	C24—H24	0.9300
C5—N2	1.378 (3)	C25—H25	0.9300
C5—C6	1.407 (3)	C26—O1	1.2140 (19)
C6—N1	1.382 (2)	C26—C27	1.466 (2)
C7—N1	1.303 (2)	C27—C28	1.426 (2)
C7—C8	1.420 (2)	C27—C31	1.438 (2)
C7—C11	1.534 (2)	C27—Fe1	2.0181 (15)
C8—N2	1.311 (2)	C28—C29	1.412 (3)
C8—C9	1.459 (3)	C28—Fe1	2.0321 (17)
C9—C15	1.391 (3)	C28—H28	0.9300
C9—C10	1.396 (2)	C29—C30	1.413 (3)
C10—C12	1.380 (2)	C29—Fe1	2.0513 (19)
C10—C11	1.527 (2)	C29—H29	0.9300
C11—N3	1.466 (2)	C30—C31	1.402 (3)
C11—C16	1.572 (2)	C30—Fe1	2.053 (2)
C12—C13	1.385 (3)	C30—H30	0.9300
C12—H12	0.9300	C31—Fe1	2.0372 (17)
C13—C14	1.382 (3)	C31—H31	0.9300
C13—H13	0.9300	C32—C33	1.400 (3)
C14—C15	1.373 (3)	C32—C36	1.396 (4)
C14—H14	0.9300	C32—Fe1	2.029 (2)
C15—H15	0.9300	C32—H32	0.9300
C16—C26	1.519 (2)	C33—C34	1.371 (4)
C16—C17	1.546 (2)	C33—Fe1	2.037 (2)
C16—H16	0.9800	C33—H33	0.9300
C17—C18	1.516 (2)	C34—C35	1.375 (4)
C17—C20	1.516 (2)	C34—Fe1	2.033 (2)
C17—H17	0.9800	C34—H34	0.9300
C18—N3	1.453 (2)	C35—C36	1.382 (5)
C18—H18A	0.9700	C35—Fe1	2.016 (2)
C18—H18B	0.9700	C35—H35	0.9300
C19—N3	1.452 (2)	C36—Fe1	2.019 (2)
C19—H19A	0.9600	C36—H36	0.9300
C19—H19B	0.9600	C37—H37A	0.9600
C19—H19C	0.9600	C37—H37B	0.9600
C20—C21	1.381 (2)	C37—H37C	0.9600
			
C2—C1—C6	119.8 (2)	C30—C29—C28	108.45 (16)
C2—C1—H1	120.1	C30—C29—Fe1	69.90 (11)
C6—C1—H1	120.1	C28—C29—Fe1	69.05 (10)
C1—C2—C3	120.3 (2)	C30—C29—H29	125.8
C1—C2—H2	119.8	C28—C29—H29	125.8
C3—C2—H2	119.8	Fe1—C29—H29	126.9
C4—C3—C2	121.2 (2)	C31—C30—C29	108.52 (16)
C4—C3—H3	119.4	C31—C30—Fe1	69.37 (10)
C2—C3—H3	119.4	C29—C30—Fe1	69.81 (11)
C3—C4—C5	119.9 (2)	C31—C30—H30	125.7
C3—C4—H4	120.0	C29—C30—H30	125.7
C5—C4—H4	120.0	Fe1—C30—H30	126.7
N2—C5—C6	122.42 (16)	C30—C31—C27	107.94 (15)
N2—C5—C4	118.8 (2)	C30—C31—Fe1	70.55 (11)
C6—C5—C4	118.8 (2)	C27—C31—Fe1	68.52 (9)
N1—C6—C1	118.50 (18)	C30—C31—H31	126.0
N1—C6—C5	121.66 (17)	C27—C31—H31	126.0
C1—C6—C5	119.84 (17)	Fe1—C31—H31	126.5
N1—C7—C8	123.88 (15)	C33—C32—C36	107.2 (3)
N1—C7—C11	125.63 (15)	C33—C32—Fe1	70.14 (13)
C8—C7—C11	110.48 (15)	C36—C32—Fe1	69.44 (15)
N2—C8—C7	123.60 (18)	C33—C32—H32	126.4
N2—C8—C9	127.79 (17)	C36—C32—H32	126.4
C7—C8—C9	108.56 (15)	Fe1—C32—H32	125.6
C15—C9—C10	121.06 (19)	C34—C33—C32	107.9 (2)
C15—C9—C8	130.39 (19)	C34—C33—Fe1	70.17 (13)
C10—C9—C8	108.48 (15)	C32—C33—Fe1	69.59 (13)
C12—C10—C9	119.76 (17)	C34—C33—H33	126.0
C12—C10—C11	128.28 (16)	C32—C33—H33	126.0
C9—C10—C11	111.88 (15)	Fe1—C33—H33	125.8
N3—C11—C10	110.80 (12)	C33—C34—C35	108.7 (3)
N3—C11—C7	116.62 (13)	C33—C34—Fe1	70.45 (13)
C10—C11—C7	100.53 (13)	C35—C34—Fe1	69.48 (14)
N3—C11—C16	102.27 (12)	C33—C34—H34	125.6
C10—C11—C16	117.61 (13)	C35—C34—H34	125.6
C7—C11—C16	109.73 (12)	Fe1—C34—H34	126.0
C10—C12—C13	118.9 (2)	C34—C35—C36	108.3 (3)
C10—C12—H12	120.6	C34—C35—Fe1	70.82 (15)
C13—C12—H12	120.6	C36—C35—Fe1	70.11 (14)
C14—C13—C12	121.1 (2)	C34—C35—H35	125.8
C14—C13—H13	119.5	C36—C35—H35	125.8
C12—C13—H13	119.5	Fe1—C35—H35	124.8
C15—C14—C13	120.8 (2)	C35—C36—C32	107.8 (3)
C15—C14—H14	119.6	C35—C36—Fe1	69.84 (16)
C13—C14—H14	119.6	C32—C36—Fe1	70.21 (14)
C14—C15—C9	118.4 (2)	C35—C36—H36	126.1
C14—C15—H15	120.8	C32—C36—H36	126.1
C9—C15—H15	120.8	Fe1—C36—H36	125.4
C26—C16—C17	114.62 (12)	C23—C37—H37A	109.5
C26—C16—C11	111.53 (12)	C23—C37—H37B	109.5
C17—C16—C11	105.82 (12)	H37A—C37—H37B	109.5
C26—C16—H16	108.2	C23—C37—H37C	109.5
C17—C16—H16	108.2	H37A—C37—H37C	109.5
C11—C16—H16	108.2	H37B—C37—H37C	109.5
C18—C17—C20	114.90 (14)	C7—N1—C6	114.35 (15)
C18—C17—C16	103.84 (12)	C8—N2—C5	114.00 (17)
C20—C17—C16	112.60 (13)	C19—N3—C18	115.01 (14)
C18—C17—H17	108.4	C19—N3—C11	116.54 (13)
C20—C17—H17	108.4	C18—N3—C11	107.65 (12)
C16—C17—H17	108.4	C35—Fe1—C27	158.95 (12)
N3—C18—C17	103.35 (13)	C35—Fe1—C36	40.05 (13)
N3—C18—H18A	111.1	C27—Fe1—C36	123.19 (11)
C17—C18—H18A	111.1	C35—Fe1—C32	67.39 (13)
N3—C18—H18B	111.1	C27—Fe1—C32	108.14 (9)
C17—C18—H18B	111.1	C36—Fe1—C32	40.35 (12)
H18A—C18—H18B	109.1	C35—Fe1—C34	39.71 (12)
N3—C19—H19A	109.5	C27—Fe1—C34	159.51 (11)
N3—C19—H19B	109.5	C36—Fe1—C34	66.96 (12)
H19A—C19—H19B	109.5	C32—Fe1—C34	66.95 (11)
N3—C19—H19C	109.5	C35—Fe1—C28	122.02 (11)
H19A—C19—H19C	109.5	C27—Fe1—C28	41.22 (6)
H19B—C19—H19C	109.5	C36—Fe1—C28	105.92 (10)
C21—C20—C25	117.41 (16)	C32—Fe1—C28	121.56 (9)
C21—C20—C17	121.82 (15)	C34—Fe1—C28	158.70 (11)
C25—C20—C17	120.75 (15)	C35—Fe1—C33	66.84 (11)
C20—C21—C22	121.10 (18)	C27—Fe1—C33	124.02 (9)
C20—C21—H21	119.5	C36—Fe1—C33	67.40 (11)
C22—C21—H21	119.5	C32—Fe1—C33	40.27 (10)
C23—C22—C21	121.60 (18)	C34—Fe1—C33	39.38 (11)
C23—C22—H22	119.2	C28—Fe1—C33	158.62 (10)
C21—C22—H22	119.2	C35—Fe1—C31	157.57 (13)
C22—C23—C24	117.38 (17)	C27—Fe1—C31	41.54 (6)
C22—C23—C37	122.12 (18)	C36—Fe1—C31	161.66 (13)
C24—C23—C37	120.50 (19)	C32—Fe1—C31	126.18 (10)
C23—C24—C25	121.52 (18)	C34—Fe1—C31	123.94 (11)
C23—C24—H24	119.2	C28—Fe1—C31	69.01 (7)
C25—C24—H24	119.2	C33—Fe1—C31	110.55 (9)
C24—C25—C20	120.98 (17)	C35—Fe1—C29	106.73 (11)
C24—C25—H25	119.5	C27—Fe1—C29	68.61 (7)
C20—C25—H25	119.5	C36—Fe1—C29	120.34 (11)
O1—C26—C27	121.96 (15)	C32—Fe1—C29	156.28 (11)
O1—C26—C16	121.68 (14)	C34—Fe1—C29	124.16 (10)
C27—C26—C16	116.33 (13)	C28—Fe1—C29	40.44 (7)
C28—C27—C31	107.21 (14)	C33—Fe1—C29	160.65 (10)
C28—C27—C26	125.44 (14)	C31—Fe1—C29	67.96 (7)
C31—C27—C26	126.87 (14)	C35—Fe1—C30	121.88 (13)
C28—C27—Fe1	69.92 (9)	C27—Fe1—C30	68.69 (7)
C31—C27—Fe1	69.94 (9)	C36—Fe1—C30	156.05 (13)
C26—C27—Fe1	119.20 (10)	C32—Fe1—C30	162.39 (11)
C29—C28—C27	107.88 (16)	C34—Fe1—C30	109.57 (10)
C29—C28—Fe1	70.51 (10)	C28—Fe1—C30	68.26 (8)
C27—C28—Fe1	68.86 (9)	C33—Fe1—C30	126.09 (10)
C29—C28—H28	126.1	C31—Fe1—C30	40.09 (7)
C27—C28—H28	126.1	C29—Fe1—C30	40.29 (8)
Fe1—C28—H28	126.1		
			
C6—C1—C2—C3	0.3 (3)	C26—C27—Fe1—C36	−44.61 (18)
C1—C2—C3—C4	1.8 (3)	C28—C27—Fe1—C32	117.50 (13)
C2—C3—C4—C5	−2.0 (3)	C31—C27—Fe1—C32	−124.53 (13)
C3—C4—C5—N2	−177.95 (19)	C26—C27—Fe1—C32	−2.69 (16)
C3—C4—C5—C6	0.1 (3)	C28—C27—Fe1—C34	−170.0 (3)
C2—C1—C6—N1	177.18 (17)	C31—C27—Fe1—C34	−52.0 (3)
C2—C1—C6—C5	−2.2 (3)	C26—C27—Fe1—C34	69.9 (3)
N2—C5—C6—N1	0.6 (3)	C31—C27—Fe1—C28	117.96 (14)
C4—C5—C6—N1	−177.38 (16)	C26—C27—Fe1—C28	−120.19 (16)
N2—C5—C6—C1	179.95 (16)	C28—C27—Fe1—C33	159.12 (12)
C4—C5—C6—C1	2.0 (3)	C31—C27—Fe1—C33	−82.91 (13)
N1—C7—C8—N2	1.3 (3)	C26—C27—Fe1—C33	38.93 (16)
C11—C7—C8—N2	−179.79 (15)	C28—C27—Fe1—C31	−117.96 (14)
N1—C7—C8—C9	178.99 (15)	C26—C27—Fe1—C31	121.85 (16)
C11—C7—C8—C9	−2.10 (18)	C28—C27—Fe1—C29	−37.51 (11)
N2—C8—C9—C15	1.2 (3)	C31—C27—Fe1—C29	80.46 (11)
C7—C8—C9—C15	−176.42 (19)	C26—C27—Fe1—C29	−157.70 (14)
N2—C8—C9—C10	178.05 (17)	C28—C27—Fe1—C30	−80.91 (11)
C7—C8—C9—C10	0.48 (19)	C31—C27—Fe1—C30	37.05 (10)
C15—C9—C10—C12	1.7 (3)	C26—C27—Fe1—C30	158.90 (14)
C8—C9—C10—C12	−175.58 (16)	C32—C36—Fe1—C35	−118.5 (2)
C15—C9—C10—C11	178.59 (16)	C35—C36—Fe1—C27	−162.74 (16)
C8—C9—C10—C11	1.34 (19)	C32—C36—Fe1—C27	78.74 (17)
C12—C10—C11—N3	50.2 (2)	C35—C36—Fe1—C32	118.5 (2)
C9—C10—C11—N3	−126.35 (15)	C35—C36—Fe1—C34	37.51 (18)
C12—C10—C11—C7	174.18 (17)	C32—C36—Fe1—C34	−81.01 (18)
C9—C10—C11—C7	−2.42 (17)	C35—C36—Fe1—C28	−121.17 (17)
C12—C10—C11—C16	−66.8 (2)	C32—C36—Fe1—C28	120.32 (16)
C9—C10—C11—C16	116.57 (16)	C35—C36—Fe1—C33	80.39 (19)
N1—C7—C11—N3	−58.6 (2)	C32—C36—Fe1—C33	−38.13 (16)
C8—C7—C11—N3	122.49 (15)	C35—C36—Fe1—C31	167.7 (2)
N1—C7—C11—C10	−178.43 (15)	C32—C36—Fe1—C31	49.2 (4)
C8—C7—C11—C10	2.68 (16)	C35—C36—Fe1—C29	−79.76 (19)
N1—C7—C11—C16	57.0 (2)	C32—C36—Fe1—C29	161.73 (14)
C8—C7—C11—C16	−121.89 (14)	C35—C36—Fe1—C30	−49.0 (3)
C9—C10—C12—C13	−2.1 (3)	C32—C36—Fe1—C30	−167.52 (19)
C11—C10—C12—C13	−178.46 (18)	C33—C32—Fe1—C35	−80.36 (18)
C10—C12—C13—C14	1.2 (3)	C36—C32—Fe1—C35	37.77 (17)
C12—C13—C14—C15	0.2 (4)	C33—C32—Fe1—C27	121.61 (15)
C13—C14—C15—C9	−0.7 (3)	C36—C32—Fe1—C27	−120.26 (17)
C10—C9—C15—C14	−0.2 (3)	C33—C32—Fe1—C36	−118.1 (2)
C8—C9—C15—C14	176.3 (2)	C33—C32—Fe1—C34	−37.11 (16)
N3—C11—C16—C26	−112.15 (13)	C36—C32—Fe1—C34	81.01 (19)
C10—C11—C16—C26	9.44 (19)	C33—C32—Fe1—C28	164.92 (14)
C7—C11—C16—C26	123.43 (14)	C36—C32—Fe1—C28	−76.96 (19)
N3—C11—C16—C17	13.11 (15)	C36—C32—Fe1—C33	118.1 (2)
C10—C11—C16—C17	134.70 (14)	C33—C32—Fe1—C31	79.02 (17)
C7—C11—C16—C17	−111.31 (14)	C36—C32—Fe1—C31	−162.85 (16)
C26—C16—C17—C18	134.85 (13)	C33—C32—Fe1—C29	−160.4 (2)
C11—C16—C17—C18	11.52 (15)	C36—C32—Fe1—C29	−42.3 (3)
C26—C16—C17—C20	−100.24 (15)	C33—C32—Fe1—C30	45.0 (4)
C11—C16—C17—C20	136.43 (13)	C36—C32—Fe1—C30	163.1 (3)
C20—C17—C18—N3	−155.76 (13)	C33—C34—Fe1—C35	119.8 (3)
C16—C17—C18—N3	−32.34 (16)	C33—C34—Fe1—C27	−42.2 (4)
C18—C17—C20—C21	−125.50 (18)	C35—C34—Fe1—C27	−162.0 (3)
C16—C17—C20—C21	115.89 (18)	C33—C34—Fe1—C36	82.0 (2)
C18—C17—C20—C25	55.8 (2)	C35—C34—Fe1—C36	−37.8 (2)
C16—C17—C20—C25	−62.8 (2)	C33—C34—Fe1—C32	37.94 (17)
C25—C20—C21—C22	1.3 (3)	C35—C34—Fe1—C32	−81.8 (2)
C17—C20—C21—C22	−177.46 (18)	C33—C34—Fe1—C28	156.3 (2)
C20—C21—C22—C23	−1.6 (3)	C35—C34—Fe1—C28	36.5 (4)
C21—C22—C23—C24	0.9 (3)	C35—C34—Fe1—C33	−119.8 (3)
C21—C22—C23—C37	−178.43 (19)	C33—C34—Fe1—C31	−81.20 (18)
C22—C23—C24—C25	−0.1 (3)	C35—C34—Fe1—C31	159.02 (19)
C37—C23—C24—C25	179.34 (18)	C33—C34—Fe1—C29	−166.04 (14)
C23—C24—C25—C20	−0.2 (3)	C35—C34—Fe1—C29	74.2 (2)
C21—C20—C25—C24	−0.4 (3)	C33—C34—Fe1—C30	−123.52 (16)
C17—C20—C25—C24	178.36 (16)	C35—C34—Fe1—C30	116.7 (2)
C17—C16—C26—O1	−26.9 (2)	C29—C28—Fe1—C35	77.86 (18)
C11—C16—C26—O1	93.32 (17)	C27—C28—Fe1—C35	−163.05 (16)
C17—C16—C26—C27	154.99 (13)	C29—C28—Fe1—C27	−119.08 (15)
C11—C16—C26—C27	−84.80 (15)	C29—C28—Fe1—C36	118.36 (16)
O1—C26—C27—C28	1.6 (2)	C27—C28—Fe1—C36	−122.55 (15)
C16—C26—C27—C28	179.68 (14)	C29—C28—Fe1—C32	159.35 (14)
O1—C26—C27—C31	172.55 (16)	C27—C28—Fe1—C32	−81.57 (15)
C16—C26—C27—C31	−9.3 (2)	C29—C28—Fe1—C34	51.2 (3)
O1—C26—C27—Fe1	86.68 (17)	C27—C28—Fe1—C34	170.3 (3)
C16—C26—C27—Fe1	−95.21 (14)	C29—C28—Fe1—C33	−173.2 (2)
C31—C27—C28—C29	−0.34 (19)	C27—C28—Fe1—C33	−54.1 (3)
C26—C27—C28—C29	172.13 (15)	C29—C28—Fe1—C31	−80.23 (12)
Fe1—C27—C28—C29	59.96 (13)	C27—C28—Fe1—C31	38.85 (10)
C31—C27—C28—Fe1	−60.29 (11)	C27—C28—Fe1—C29	119.08 (15)
C26—C27—C28—Fe1	112.17 (15)	C29—C28—Fe1—C30	−37.05 (11)
C27—C28—C29—C30	0.0 (2)	C27—C28—Fe1—C30	82.03 (11)
Fe1—C28—C29—C30	58.96 (14)	C34—C33—Fe1—C35	−37.09 (19)
C27—C28—C29—Fe1	−58.93 (12)	C32—C33—Fe1—C35	81.8 (2)
C28—C29—C30—C31	0.3 (2)	C34—C33—Fe1—C27	163.53 (16)
Fe1—C29—C30—C31	58.72 (13)	C32—C33—Fe1—C27	−77.54 (17)
C28—C29—C30—Fe1	−58.43 (13)	C34—C33—Fe1—C36	−80.7 (2)
C29—C30—C31—C27	−0.5 (2)	C32—C33—Fe1—C36	38.20 (18)
Fe1—C30—C31—C27	58.50 (11)	C34—C33—Fe1—C32	−118.9 (2)
C29—C30—C31—Fe1	−59.00 (14)	C32—C33—Fe1—C34	118.9 (2)
C28—C27—C31—C30	0.51 (19)	C34—C33—Fe1—C28	−156.4 (2)
C26—C27—C31—C30	−171.81 (15)	C32—C33—Fe1—C28	−37.4 (3)
Fe1—C27—C31—C30	−59.76 (12)	C34—C33—Fe1—C31	118.88 (17)
C28—C27—C31—Fe1	60.28 (11)	C32—C33—Fe1—C31	−122.19 (16)
C26—C27—C31—Fe1	−112.05 (15)	C34—C33—Fe1—C29	37.1 (4)
C36—C32—C33—C34	0.1 (3)	C32—C33—Fe1—C29	156.0 (3)
Fe1—C32—C33—C34	59.93 (17)	C34—C33—Fe1—C30	76.43 (19)
C36—C32—C33—Fe1	−59.82 (16)	C32—C33—Fe1—C30	−164.64 (15)
C32—C33—C34—C35	−0.4 (3)	C30—C31—Fe1—C35	−43.2 (3)
Fe1—C33—C34—C35	59.12 (18)	C27—C31—Fe1—C35	−162.6 (2)
C32—C33—C34—Fe1	−59.57 (15)	C30—C31—Fe1—C27	119.34 (15)
C33—C34—C35—C36	0.6 (3)	C30—C31—Fe1—C36	157.9 (3)
Fe1—C34—C35—C36	60.33 (18)	C27—C31—Fe1—C36	38.5 (3)
C33—C34—C35—Fe1	−59.72 (18)	C30—C31—Fe1—C32	−164.77 (13)
C34—C35—C36—C32	−0.5 (3)	C27—C31—Fe1—C32	75.89 (13)
Fe1—C35—C36—C32	60.25 (17)	C30—C31—Fe1—C34	−80.07 (15)
C34—C35—C36—Fe1	−60.78 (19)	C27—C31—Fe1—C34	160.58 (13)
C33—C32—C36—C35	0.3 (3)	C30—C31—Fe1—C28	80.78 (12)
Fe1—C32—C36—C35	−60.01 (18)	C27—C31—Fe1—C28	−38.56 (9)
C33—C32—C36—Fe1	60.27 (15)	C30—C31—Fe1—C33	−122.11 (13)
C8—C7—N1—C6	−2.9 (2)	C27—C31—Fe1—C33	118.55 (12)
C11—C7—N1—C6	178.34 (14)	C30—C31—Fe1—C29	37.17 (11)
C1—C6—N1—C7	−177.38 (15)	C27—C31—Fe1—C29	−82.17 (11)
C5—C6—N1—C7	2.0 (2)	C27—C31—Fe1—C30	−119.34 (15)
C7—C8—N2—C5	1.4 (2)	C30—C29—Fe1—C35	119.88 (16)
C9—C8—N2—C5	−175.84 (16)	C28—C29—Fe1—C35	−120.06 (16)
C6—C5—N2—C8	−2.2 (2)	C30—C29—Fe1—C27	−81.86 (12)
C4—C5—N2—C8	175.73 (17)	C28—C29—Fe1—C27	38.20 (10)
C17—C18—N3—C19	175.16 (14)	C30—C29—Fe1—C36	161.28 (16)
C17—C18—N3—C11	43.41 (16)	C28—C29—Fe1—C36	−78.66 (17)
C10—C11—N3—C19	68.04 (18)	C30—C29—Fe1—C32	−168.4 (2)
C7—C11—N3—C19	−46.10 (19)	C28—C29—Fe1—C32	−48.4 (3)
C16—C11—N3—C19	−165.80 (14)	C30—C29—Fe1—C34	79.95 (16)
C10—C11—N3—C18	−161.05 (13)	C28—C29—Fe1—C34	−159.99 (14)
C7—C11—N3—C18	84.81 (16)	C30—C29—Fe1—C28	−120.06 (16)
C16—C11—N3—C18	−34.89 (15)	C30—C29—Fe1—C33	52.4 (3)
C34—C35—Fe1—C27	162.4 (2)	C28—C29—Fe1—C33	172.5 (2)
C36—C35—Fe1—C27	43.7 (4)	C30—C29—Fe1—C31	−36.99 (11)
C34—C35—Fe1—C36	118.7 (3)	C28—C29—Fe1—C31	83.07 (11)
C34—C35—Fe1—C32	80.7 (2)	C28—C29—Fe1—C30	120.06 (16)
C36—C35—Fe1—C32	−38.04 (17)	C31—C30—Fe1—C35	162.08 (14)
C36—C35—Fe1—C34	−118.7 (3)	C29—C30—Fe1—C35	−77.92 (17)
C34—C35—Fe1—C28	−165.24 (16)	C31—C30—Fe1—C27	−38.35 (10)
C36—C35—Fe1—C28	76.0 (2)	C29—C30—Fe1—C27	81.65 (12)
C34—C35—Fe1—C33	36.79 (18)	C31—C30—Fe1—C36	−163.0 (2)
C36—C35—Fe1—C33	−81.92 (19)	C29—C30—Fe1—C36	−43.0 (3)
C34—C35—Fe1—C31	−51.2 (4)	C31—C30—Fe1—C32	44.5 (3)
C36—C35—Fe1—C31	−169.9 (2)	C29—C30—Fe1—C32	164.5 (3)
C34—C35—Fe1—C29	−123.77 (19)	C31—C30—Fe1—C34	119.85 (14)
C36—C35—Fe1—C29	117.52 (18)	C29—C30—Fe1—C34	−120.15 (14)
C34—C35—Fe1—C30	−82.4 (2)	C31—C30—Fe1—C28	−82.81 (11)
C36—C35—Fe1—C30	158.85 (17)	C29—C30—Fe1—C28	37.19 (11)
C28—C27—Fe1—C35	43.5 (3)	C31—C30—Fe1—C33	78.96 (15)
C31—C27—Fe1—C35	161.4 (3)	C29—C30—Fe1—C33	−161.03 (13)
C26—C27—Fe1—C35	−76.7 (3)	C29—C30—Fe1—C31	120.00 (16)
C28—C27—Fe1—C36	75.58 (16)	C31—C30—Fe1—C29	−120.00 (16)
C31—C27—Fe1—C36	−166.46 (14)		

### Hydrogen-bond geometry (Å, º)

**Table:**

*D*—H···*A*	*D*—H	H···*A*	*D*···*A*	*D*—H···*A*
C28—H28···N3^i^	0.93	2.49	3.416 (2)	176

Symmetry code: (i) −*x*+1/2, *y*−1/2, −*z*+1/2.
